# Transition from fossil fuels to nuclear and renewable energy: Evidence from Japan

**DOI:** 10.1371/journal.pone.0353734

**Published:** 2026-11-13

**Authors:** William M. Cheung

**Affiliations:** Waseda Business School, Faculty of Commerce, Governance & Sustainability, Research Institute (GSRI), Waseda University, Tokyo, Japan; Shanghai Ocean University, CHINA

## Abstract

Japan has pledged carbon neutrality by 2050, yet energy production still accounts for 84% of its greenhouse-gas emissions, and only 33.4% (328.48 TWh) of its 2023 electricity generation came from nuclear and renewable sources. This paper asks how Japan’s electricity generation can complete the transition from fossil fuels to nuclear and renewable energy. Using annual data for 1980–2023 from the World Bank, IMF, and the Statistics Bureau of Japan, we estimate an autoregressive log-growth model of electricity consumption driven by GDP per capita, median age, and population change, and project Japan’s 2050 electricity consumption at approximately 1,000 TWh (999 TWh; range 900–1,000 TWh across specifications, sample horizons, and population paths). Closing the resulting clean-energy gap of roughly 469 TWh would require the equivalent of about 56 additional nuclear reactors of average current capability (8.41 TWh each), rising to about 67 at the capacity factors Japan has realized since Fukushima. Applying machine-learning facility-location methods (genetic and NSGA-II algorithms) to a benchmark 21-plant configuration, we show that siting new plants on Japan’s least-populated coastlines reduces the population inside their emergency planning zones by roughly two orders of magnitude relative to naive placement, at an additional transmission cost of a few hundred million US dollars per year. Public acceptance, rather than engineering capacity, is the binding constraint on Japan’s nuclear future, and siting policy that takes safety over efficiency directly addresses it. Hydrogen-based generation, whose commercial viability at scale by 2050 remains unconfirmed, lies outside the scope of the quantitative analysis.

## 1. Introduction

*“Japan prepares to restart world’s biggest nuclear plant, 15 years after Fukushima”, Reuters December 22, 2025.*

From the 1997 Kyoto Protocol to the 2021 Paris Agreement, the Japanese government has expressed a strong determination to address climate change at the national level, pledging to achieve carbon neutrality by 2050. Japan has also promised to provide financial and technical assistance to developing countries, supporting them in developing their own net-zero strategies and initiatives. In doing so, Japan seeks to strengthen its role as a global leader on climate and environmental issues and to support global efforts to combat climate change [1].

While the goal of carbon neutrality by 2050 is clear, the path to achieving it is not—or at least not yet. For Japan, the single largest source of carbon emissions is energy production, which accounts for 84% of the country’s greenhouse gas (GHG) emissions, a share that can be tracked in the annually updated National GHG Inventory Document of Japan [2,3]. To achieve carbon neutrality by 2050, the Japanese government must find a feasible way to generate electricity from sustainable fuels rather than fossil fuels. In this paper, we ask one fundamental question: how can Japan’s electricity generation transition from fossil fuels to sustainable fuels? Specifically, what are the feasible pathways for (1) replacing fossil fuels with sustainable fuels in electricity generation and achieving carbon neutrality between now and 2050, and (2) remaining sustainable from that point onward?

At present, fossil fuels are a major part of Japan’s energy mix. As of 2022, the country’s dependence on fossil fuels in its primary energy supply had risen to 83.5% [4]. This reliance not only makes Japan one of the largest greenhouse gas emitters among developed countries but also underscores the urgent need to restructure its energy sector strategically. Electricity generation in Japan has long been dominated by fossil fuels, and the Fukushima Daiichi nuclear disaster in 2011 further entrenched this dependence. After 2011, Japan suspended all of its nuclear power plants over safety concerns, increasing its reliance on fossil fuels such as coal, natural gas, and oil. The combustion of fossil fuels is a major source of carbon dioxide (CO_2_) and other greenhouse gases that contribute directly to global warming and climate change. Other pollutants released by combustion, such as sulfur dioxide (SO_2_) and nitrogen oxides (NO_x_), also degrade air quality and can harm public health.

Beyond the carbon-neutrality pledge and public health concerns, the cost of importing fossil fuels places another significant burden on the Japanese economy. In FY2023 alone, fossil fuel imports cost JPY 27.3 trillion (4.14% of 2023 GDP). Between 2010 and 2023, Japan imported roughly 400–500 million tons of fossil fuels annually—mainly coal and natural gas—at an average cost of JPY 20 trillion. From 2020 to 2022, the value of these imports rose by JPY 22.4 trillion, from JPY 11.3 trillion to JPY 33.7 trillion, contributing to an outflow of national wealth and the largest trade deficit in the country’s history (JPY 20.3 trillion). The underlying series are published in the Ministry of Finance Trade Statistics of Japan [5], and the energy-import figures are reported in the METI energy statistics [6].

Using a simple log-growth model [7–9], we estimate the factor loadings of GDP growth, population age, and change in population size for one-year-ahead electricity consumption. Combining these loadings with projections of the same covariates for 2050 from the World Bank, ESCAP, and PwC, we project Japan’s 2050 electricity consumption at approximately 1,000 TWh (999 TWh under the World Bank population projection). Given that Japan’s existing nuclear (277.5 TWh) and renewable (252.75 TWh) capacity could supply 530 TWh once all 33 operable reactors are restarted, we define the clean energy gap as the difference between projected 2050 consumption and current capacity, which equals 469 TWh. We take nuclear power as the lead technology for closing this gap because it is a stable technology with global adoption. Unlike solar and wind, whose output fluctuates with weather and daylight, nuclear plants deliver continuous baseload electricity: the world’s reactor fleet ran at an average capacity factor of 83% in 2024, higher than any other source of electricity [10]. Nor is the technology unproven—416 commercial power reactors were in operation across 31 countries as of 2025 [11], and France, the most prominent adopter, generated 67% of its electricity from nuclear power in 2024, the highest share in the world [12]. This stability means the required capacity can be counted directly in reactors. This is equivalent to the capacity of approximately 56 new nuclear reactors, each matching the 8.41 TWh average output capability of the current 33 operable reactors—or up to about 67 reactors once realized post-Fukushima capacity factors are taken into account (Section 5.3). Whether the required reactors can be built before 2050 depends less on construction engineering than on licensing throughput, supply-chain capacity, and the nuclear workforce (Section 5.3). The greater hurdle is gaining public trust in the safety of nuclear plants. Above all, TEPCO and the Japanese government must work together to ensure risk-management practices robust enough to keep the Fukushima tragedy firmly in the past.

This paper also adds a spatial dimension to the transition debate. The number of reactors needed is only half of the policy problem; the other half is where to build them, because the dominant societal cost of nuclear power—the population placed at risk by a severe accident—depends almost entirely on siting. We treat the location of new plants as an obnoxious facility-location problem and solve it with evolutionary optimization: a genetic algorithm that minimizes the population inside the plants’ emergency planning zones, and a multi-objective NSGA-II formulation that traces the trade-off between this exposure and the cost of transmitting power from remote sites to demand centres. The results show that the societal risk of nuclear expansion is not a fixed quantity but a design variable: optimized coastal siting reduces the exposed population by roughly two orders of magnitude, while the associated transmission penalty is modest. On this basis we argue that siting policy should take safety over efficiency—accepting a small, quantifiable supply cost in exchange for a large reduction in the population at risk, which is precisely the bargain most likely to rebuild public trust.

Two considerations motivate this study. First, while Japan’s official energy planning documents set detailed targets for 2030 [6], the quantitative arithmetic of the full 2050 transition—how much electricity will be demanded, how large the clean-energy gap is once existing nuclear and renewable capability is exhausted, and how many new reactors that gap represents—has received far less systematic attention. Existing academic studies of Japan’s 2050 energy mix are predominantly supply-side optimization exercises that take future demand as a scenario input: Ōtsuki et al. [13] minimize system cost subject to siting constraints on variable renewables, Ōtsuki et al. [14] quantify the marginal cost of a fully renewable power supply, and Cheng et al. [15] demonstrate the technical feasibility of 100% renewable electricity with storage—in each case conditional on an assumed demand path [13–15]. A transparent, replicable demand-side benchmark, estimated from Japan’s own consumption history and accompanied by explicit diagnostic testing, is a necessary complement to that literature: every downstream quantity in the transition debate, from reactor counts to land-use requirements, scales with projected demand. Second, the policy debate after Fukushima has concentrated on whether to expand nuclear power, while the equally consequential question of where to put new plants—which largely determines the population placed at risk by a severe accident—has been treated informally. Quantifying the trade-off between siting safety and supply cost speaks directly to the public-trust deficit that this paper identifies as the binding constraint on Japan’s nuclear future.

This study makes three contributions. First, it provides an econometric benchmark of Japan’s 2050 electricity demand: an autoregressive log-growth model estimated on 1980–2023 data, supported by unit-root and cointegration tests, autocorrelation diagnostics with robust standard errors, and robustness checks that add energy prices, industrial structure, urbanization, and the generation mix to the baseline specification. Second, it translates the projected demand into policy-legible units—terawatt-hours of clean-energy gap and reactor-equivalents—with an explicit sensitivity analysis to the capacity factors Japan’s fleet has actually realized since Fukushima. Third, it treats reactor siting as a design variable rather than a fixed cost: using evolutionary multi-objective optimization, it quantifies the Pareto frontier between the population exposed in emergency planning zones and the cost of transmitting power from remote coastal sites, showing that societal risk can be reduced by roughly two orders of magnitude at modest supply cost. To our knowledge, this is the first application of obnoxious facility-location optimization to Japan’s post-Fukushima nuclear siting debate.

The remainder of the paper is organized as follows. Section 2 reviews Japan’s current energy landscape. Section 3 describes the data and the econometric methodology. Section 4 presents the empirical results, diagnostic tests, and the projection of Japan’s electricity consumption in 2050. Section 5 examines the potential of nuclear and renewable energy to replace fossil fuels, estimates the number of new reactors required to close the resulting energy gap under alternative scenarios, and analyzes where new reactors could be sited to minimize the population exposed to a potential nuclear emergency. Section 6 discusses the findings in relation to the existing literature, and Section 7 concludes with specific policy recommendations.

## 2. Japan’s current energy landscape

### 2.1. Developments of Japan’s energy after world war II

*“福島第一原発事故は、改めて原発が抱えるリスクを人びとに再認識させた。「再生可能エネルギーの開発を進めるべきだ」という意見を8割近くが支持しており, 「原発ゼロを目指すべきだ」を5割近く, 「原発の再稼働を進めるべきではない」も4割強が支持している。反対に, 「脱原発は現実的ではない」も4割弱いる。原発に反対するにせよ賛成するにせよ, 「福島原発事故は収束した」と考えるのは数%の人しかいない。” 遠藤薫, 2021*

*[English translation: “The Fukushima Daiichi nuclear accident made people recognize anew the risks that nuclear power carries. Nearly 80% support the view that ‘the development of renewable energy should be advanced’; close to 50% support ‘we should aim for zero nuclear power,’ and a little over 40% support ‘the restart of nuclear reactors should not be promoted.’ Conversely, just under 40% hold that ‘phasing out nuclear power is not realistic.’ Whether they oppose or support nuclear power, only a few percent of people believe that ‘the Fukushima nuclear accident has been brought to a close.’” — Endō Kaoru, 2021] [* 16 *]*

Over the years, Japan’s energy mix has undergone significant transformations, shaped by shifts in policy, economic conditions, technological progress, and external events. Japan’s energy strategy has been defined by its limited domestic resources, which create a heavy reliance on imported energy. Over the long run, this dependence has shaped Japan’s energy structure and policies across different periods, from the post-World War II industrialization era to the present day.

In the early postwar period, Japan’s electricity generation depended heavily on coal. The rapid industrial expansion of the 1950s and 1960s made coal the backbone of Japan’s energy supply, underpinning its manufacturing industries and rapid economic growth. During this period, coal accounted for more than half of the country’s total electricity generation, while the use of heavy oil also rose dramatically [17].

In the 1970s, Japan’s energy landscape changed significantly because of the global oil crises. The 1973 oil embargo and the 1979 Iranian Revolution caused severe disruptions to global oil supply and sharp price increases. These crises exposed the vulnerability of Japan’s heavy dependence on oil imports, which made up more than 75% of its energy mix by the early 1970s [4]. In response, Japan launched an energy-diversification strategy aimed at reducing oil dependency and enhancing energy security.

Nuclear power emerged in the 1970s as a result of this strategy. Japan invested heavily in nuclear technology and built nuclear power reactors, viewing nuclear energy as a reliable and domestically manageable source that could reduce its dependence on imported oil. By the time of the Great East Japan Earthquake in 2011, nuclear energy accounted for nearly 30% of Japan’s total energy mix, and its share appeared set to keep growing. This expansion was supported by numerous public policies that promoted nuclear development [18].

At the same time, Japan invested in natural gas as a cleaner alternative to coal and oil. The development of liquefied natural gas (LNG) technology in the 1960s allowed Japan to import gas efficiently from distant regions. Although LNG is still a fossil fuel that emits greenhouse gases when used to generate electricity, it offers lower costs and reduced CO2 emissions relative to coal and oil. Throughout the 1980s and 1990s, Japan’s LNG imports continued to grow, making gas a significant share of the energy mix. By the start of this century, natural gas accounted for roughly 20% of energy consumption, used mainly for electricity generation and household heating [6].

It is worth noting that the share of renewable energy sources—such as hydro, solar, wind, and geothermal—remained relatively low until the early 2000s, although hydroelectricity maintained a consistent presence, contributing around 5–10% of electricity generation [6]. Compared with other energy sources, renewables faced several obstacles, including geographical limitations, high costs, technological barriers, and limited policy support.

The devastating earthquake and tsunami of March 11, 2011, which triggered the nuclear disaster at the Fukushima Daiichi power plant, had profound effects on Japan’s energy policy and subsequent energy mix. The crisis led to the shutdown of every nuclear reactor in the country, dramatically altering its energy landscape. As a result, Japan’s reliance on fossil fuels—particularly liquefied natural gas and coal—surged to compensate for the lost nuclear capacity. By 2013, natural gas represented nearly 40% of electricity generation and coal about 28–30% [19]. Driven by this increased fossil fuel use, Japan’s carbon emissions rose sharply and energy costs climbed, both of which challenged the country’s environmental and economic objectives.

After the Fukushima disaster and the suspension of all nuclear reactors, Japan revisited its renewable energy strategy. The Feed-in Tariff (FiT) system, introduced in 2012, aimed to accelerate the adoption of renewable energy. It marked a milestone in Japan’s energy transition: by guaranteeing a fixed price for electricity generated from renewable sources, it encouraged significant investment in solar power [20]. Wind energy, both onshore and offshore, also gained attention, though high costs and regulatory challenges slowed its progress. Hydropower, a mature and long-established source, continues to play a vital role in Japan’s energy mix, particularly through small and micro-hydro projects built on the country’s mountainous terrain [21].

In recent years, against the backdrop of the Paris Agreement, the Japanese government has set ambitious targets to reshape the energy mix by 2030 and beyond. The Strategic Energy Plan, revised periodically by the Agency for Natural Resources and Energy, sets out targets for increasing the share of renewable energy and reintroducing nuclear power under stricter safety standards. By 2030, Japan aims for renewable energy to make up 36–38% of the electricity mix and nuclear energy around 20–22%, with a corresponding reduction in fossil fuels [6].

Japan is also exploring innovative technologies such as hydrogen energy, envisioning a hydrogen-based economy by mid-century. It is recognized as one of the few countries with advanced R&D capabilities in hydrogen technology. As a versatile energy carrier, hydrogen is expected to help decarbonize industries that are difficult to electrify and to provide clean backup power. Hydrogen’s prospective role deserves an explicit justification, because it is named in Japan’s own Strategic Energy Plan, which envisages hydrogen and ammonia supplying on the order of 1% of electricity generation by 2030 [3]. Three considerations nevertheless lead us to exclude hydrogen from the quantitative 2050 electricity mix analyzed in this paper. First, hydrogen-fired generation has not yet been demonstrated at commercial scale anywhere, and the timing of its cost competitiveness is not possible to anticipate from current evidence. Second, large-scale deployment would require a dedicated import, storage, and pipeline infrastructure whose construction has not begun. Third, even the official planning trajectory implies a generation share by mid-century that is an order of magnitude smaller than the fossil-fuel share that must be replaced. We therefore treat hydrogen as a scope limitation of this study rather than as a quantified transition pathway—a limitation flagged in the abstract—and note the direction of the resulting bias: if hydrogen generation does become commercially viable before 2050, the required nuclear and renewable build-out estimated below would shrink accordingly.

According to the Institute for Sustainable Energy Policies, an independent non-profit research organization in Japan, about 28.3% of Japan’s electricity generation in 2023 came from coal, 29% from natural gas, and 9.3% from oil, so that fossil fuels accounted for approximately 66.6% of the energy mix. Renewable sources—including hydro, solar, wind, and geothermal—contributed about 25.7%, while nuclear power, slowly being restarted under stringent safety protocols, contributed approximately 7.7% [22]. This heavy reliance on fossil fuels carries significant environmental implications. As noted, the energy sector alone is responsible for a large share of the nation’s greenhouse gas emissions, so the decarbonization of electricity generation must be the top priority for meeting Japan’s carbon-neutrality commitments.

Overall, the evolution of Japan’s energy mix reflects a trajectory shaped by external circumstances, domestic demand, and advancing technological capabilities. From the postwar reliance on coal, to the strategic adoption of nuclear power, to the shift toward natural gas, and finally to the recent growth in renewables and the renewed reliance on fossil fuels after Fukushima, Japan’s energy policy has continually adapted to a changing landscape of challenges and opportunities.

### 2.2. How much energy from fossil fuels to be replaced?

Japan’s current energy landscape highlights the urgent need to transition toward cleaner energy in order to meet the country’s sustainability goals and its commitment to net-zero emissions. In 2023, Japan generated a total of 983.46 TWh of electricity [6], of which approximately 654.99 TWh came from fossil fuels (Table 1)—generation that must be replaced by a combination of nuclear and renewable energy by 2050.

**Table 1:** Size of electricity generated in 2023 by fossil fuels and sustainable fuels.

share of electricity	in TWh	Percentage
Fossil Fuel	654.99	66.6%
Renewables	252.75	25.7%
Nuclear	75.73	7.7%
subtotal	983.46	100.0%

### 2.3. Nuclear power and “S+3E” energy policy

In October 2020, Japan declared its aim of achieving carbon neutrality by 2050. The country’s policy framework and incentives for electricity generation are an indispensable part of this goal, designed to promote the development and integration of nuclear and renewable energy. Japan’s strategy takes a comprehensive approach that includes regulatory measures, financial incentives, support for innovation, and international cooperation.

In 2021, after reviewing its energy strategy in the wake of the Fukushima Daiichi disaster, the Japanese government enacted the “S+3E” energy policy, which stands for Safety, Energy Security, Economic Efficiency, and Environmental Protection [6]. Of the four, Safety is the foremost priority: the policy ensures that all energy sources, especially nuclear, comply with stricter safety regulations to prevent accidents and protect the public. Energy Security reflects Japan’s aim of maintaining a stable, reliable energy supply; because the country imports a large share of its fossil fuels, reducing the risk of supply disruptions is essential. Economic Efficiency refers to using energy in a way that supports economic growth without unnecessary cost. Finally, Environmental Protection reflects Japan’s commitment to reducing greenhouse gas emissions in line with the Paris Agreement. Each element of the “S+3E” policy is important, and together they express Japan’s choices about how it produces and uses energy.

Japan recognizes that nuclear energy offers multiple advantages as a stable, low-carbon source, which is essential for balancing the relatively variable output of renewables such as solar and wind. After the Fukushima Daiichi disaster, Japan faced enormous public and regulatory challenges in using nuclear power. Despite this setback, the government reaffirmed the indispensable role of nuclear power in the nation’s energy mix. The post-Fukushima era has been marked by comprehensive safety enhancements and regulatory reform, beginning with the establishment of the Nuclear Regulation Authority (NRA). Under the guidance of “S+3E,” the NRA has enforced rigorous safety standards and mandated extensive upgrades to existing reactors—such as improved seismic resistance and enhanced emergency-response plans—to ensure that Japan’s nuclear infrastructure can withstand future natural disasters and other risks [23].

### 2.4. Renewable energy and FiT policies

According to Japan’s 6th Strategic Energy Plan, released by the Ministry of Economy, Trade and Industry in 2021, the country aims to raise the share of renewable energy in the electricity mix to 22–24% by 2030. One of the cornerstone incentives for renewable energy development is the Feed-in Tariff (FiT) scheme, launched in July 2012. The scheme requires power companies to purchase electricity generated from certified renewable sources at fixed, government-set prices over a long-term period, so that the prospect of stable revenue encourages investment in renewable power generation. These certified sources include solar, wind, hydro, geothermal, and biomass. By providing investors with stable, predictable returns, the mechanism has effectively driven significant investment in renewable energy. As a result, Japan quickly became one of the largest solar markets in the world: by 2022, it ranked third globally in solar generation, with 9.9% of its annual electricity coming from solar energy [24].

In addition to the FiT scheme, the Japanese government provides various subsidies and tax incentives to support renewable energy development. For example, METI offers considerable subsidies for clean-energy investment, including the adoption of clean energy, energy-efficiency and conservation technologies, and decarbonization products in specific regions. Through these incentives, METI aims to reduce the initial capital costs of renewable energy installations and encourage their widespread adoption. Tax incentives—including accelerated depreciation and tax credits for investment in renewable energy systems—further improve the economic feasibility of such projects [20].

Regulatory reform also plays a pivotal role in strengthening renewable energy within Japan’s energy landscape. The government is working to establish technology standards, implement new technologies as quickly as possible, and promote innovative energy solutions. Alongside renewable deployment, energy efficiency remains a cornerstone of Japan’s strategy: the “Top Runner Program,” introduced in 1999, has set stringent energy-performance standards for energy-consuming products such as household appliances and vehicles, and continues to raise the benchmark for efficiency [25].

International cooperation and partnerships also play a key role in Japan’s transition to a sustainable energy future. Working with partner countries, Japan actively engages in global platforms such as the International Renewable Energy Agency (IRENA), the International Energy Agency (IEA), and the Paris Agreement to promote knowledge exchange, technology transfer, and collaborative R&D. The government also helps lead international discussions and multilateral negotiations by proposing rulemaking and the formulation of global standards [26], and it encourages foreign capital, direct investment, and M&A. These partnerships help Japan stay at the forefront of renewable energy innovation and implementation and support the global effort to combat climate change.

## 3. Data and methodology

### 3.1. Variable selection

Projecting Japan’s electricity consumption for 2050 is crucial for developing the country’s energy policy and planning, because it allows stakeholders—government, industry, and communities—to prepare adequately for the future and make strategic decisions about energy policy, infrastructure development, and innovation in energy production and consumption.

In analyzing the trend of Japan’s electricity consumption toward 2050, selecting appropriate variables for the regression analysis is essential for an accurate projection. Energy consumption is influenced by three primary factors: population, median age, and GDP per capita. Together, these variables provide a comprehensive framework for understanding the multifaceted drivers of electricity demand, each reflecting a distinct aspect of the social or economic dynamics that shape consumption patterns.

Population size is the fundamental determinant of a country’s electricity consumption. As the total number of people grows, overall demand for electricity tends to rise as well, owing to factors such as greater household needs, more public and commercial activity, and increased industrial production. Because Japan is widely expected to continue experiencing demographic change, understanding population trends is vital, and including them is essential when projecting electricity demand.

Median age is another critical variable, as it reflects the demographic structure of the population. Japan is known for its aging population and large share of elderly people. Median age affects electricity consumption in several ways, since older populations may have different energy-use habits from younger ones. For instance, a larger share of younger and working-age people may increase electricity use for electronic devices, especially as home-office arrangements become more common. Conversely, an aging population may reduce overall economic activity, potentially offsetting some of the rise in residential consumption. Analyzing median age therefore provides essential insight into how demographic structure influences energy demand.

GDP per capita is also critical to understanding energy trends, as it indicates a country’s economic development and personal income levels. As GDP per capita rises, households typically gain more disposable income, allowing them to purchase more energy-consuming appliances, electronics, and vehicles, which raises overall residential demand for electricity. Higher GDP per capita is also often associated with urbanization and industrial growth: as the economy expands, industrial activity tends to increase, requiring large amounts of energy. Industries—especially in manufacturing, high technology, and services—consume substantial electricity to operate efficiently.

Taken together, these three variables—population, median age, and GDP per capita—provide a comprehensive view of the drivers of Japan’s electricity consumption in 2050. Population size determines baseline demand, median age provides insight into demographic influences on consumption patterns, and GDP per capita reflects overall economic activity and its relationship with energy use. Using these variables in a regression analysis enables a thorough understanding of the complex interrelationships shaping future electricity consumption, equipping stakeholders with solid, data-driven insights for planning sustainable energy strategies.

### 3.2. The data

Several reliable data sources (Table 2) are used to conduct the regression analysis and to explore the relationship between the dependent variable (Japan’s historical annual electricity consumption) and the three independent variables (population, median age, and GDP per capita). Historical electricity consumption data are obtained from the International Energy Agency (IEA), which provides detailed accounts of global energy consumption trends. Population data are drawn from the United Nations database, which compiles demographic statistics on population change over recent decades. Median age data are taken from the Statistics Bureau of Japan, and GDP per capita data are retrieved from the International Monetary Fund (IMF) database, which provides comprehensive data for assessing Japan’s economic trends.

**Table 2:** Variables used in the linear regression analysis.

Variables	Period	Source	URL
Electricity consumption	1990-2023	World Bank Group	https://data.worldbank.org/indicator/EG.USE.ELEC.KH.PC?locations=JP
Electricity consumption	1980-1989	CountryEconomy.com	https://countryeconomy.com/energy-and-environment/electricity-consumption/japan?year=1989
Population	1980-2023	World Bank Group	https://data.worldbank.org/indicator/SP.POP.TOTL?locations=JP
Median Age	1980-2023	Population Census, Statistics Bureau of Japan	https://www.stat.go.jp/english/data/kokusei/index.html
GDP per capita	1980-2023	International Monetary Fund	https://www.imf.org/external/datamapper/NGDPD@WEO/JPN?zoom=JPN&highlight=JPN

All dependent and independent variables range from 1980 to 2023. Applying a natural-logarithmic transformation to these variables yields a more linear relationship and stabilizes the variance, which benefits the regression analysis. The data sources are summarized in Table 2, and the underlying annual series are publicly available at the URLs listed in Table 3.

**Table 3:** Variables, sample periods, and data sources.

Variables	Period	Source	URL
Electricity consumption	1990-2023	World Bank Group	https://data.worldbank.org/indicator/EG.USE.ELEC.KH.PC?locations=JP
Electricity consumption	1980-1989	CountryEconomy.com	https://countryeconomy.com/energy-and-environment/electricity-consumption/japan?year=1989
Population	1980-2023	World Bank Group	https://data.worldbank.org/indicator/SP.POP.TOTL?locations=JP
Median Age	1980-2023	Population Census, Statistics Bureau of Japan	https://www.stat.go.jp/english/data/kokusei/index.html
GDP per capita	1980-2023	International Monetary Fund	https://www.imf.org/external/datamapper/NGDPD@WEO/JPN?zoom=JPN&highlight=JPN
Consumer energy price index (used in the real energy price, Table 8)	1955–2021	OECD Main Economic Indicators, via FRED	https://fred.stlouisfed.org/series/JPNCPIENGMINMEI
All-items consumer price index (deflator for the real energy price, Table 8)	1955–2021	OECD Main Economic Indicators, via FRED	https://fred.stlouisfed.org/series/JPNCPIALLMINMEI
Urban population share (Table 8)	1980–2024	World Bank Group	https://data.worldbank.org/indicator/SP.URB.TOTL.IN.ZS?locations=JP
Industry share of value added (Table 8)	1994–2024	World Bank Group	https://data.worldbank.org/indicator/NV.IND.TOTL.ZS?locations=JP
Nuclear share of electricity generation (Table 8)	1985–2023	Ember/ Energy Institute, via Our World in Data	https://ourworldindata.org/grapher/share-electricity-nuclear
Renewable share of electricity generation (Table 8)	1985–2023	Ember/ Energy Institute, via Our World in Data	https://ourworldindata.org/grapher/share-electricity-renewables
Population projection to 2050 (Table 9)	2024–2050	World Bank population estimates and projections	https://databank.worldbank.org/source/population-estimates-and-projections
City locations and populations (Figs 2–3)	2024 snapshot	GeoNames (CC BY 4.0)	https://download.geonames.org/export/dump/cities15000.zip
Map boundaries (Figs 1–3)	—	dataofjapan/land (Global Map Japan, GSI; Public Data License v1.0)	https://github.com/dataofjapan/land

Notes on the additional series: the real energy price used in Table 8 is the annual mean of the monthly consumer energy price index divided by the annual mean of the monthly all-items index, entering the regressions in lagged logarithms; the ratio is available through 2021, matching the estimation sample. The industry share of value added begins in 1994, so its Table 8 column spans 1995–2021 after lagging. The nuclear and renewable generation shares enter as lagged fractions from 1985 (Table 8 column sample 1986–2021). The assembled analysis files and all author-generated code are provided with the submission.

### 3.3. Model specification

This section presents a simplified log-growth model of electricity consumption to examine the relationship between GDP per capita, median age, population change rate, and electricity consumption in Japan over 1980–2023 (the effective estimation sample is 1982–2021 once lagged regressors and the availability of interpolated median age are taken into account). Observations on consumption, population, and GDP per capita through 2024 are available in the source databases and are used in an extended-sample robustness check (Section 4.3); median age is interpolated between quinquennial censuses and therefore ends with the 2020 Census, and 2025 values of the source series had not yet been published at the time of writing. The aim is to understand how these factors interact and contribute to the overall trend in electricity consumption, and to project Japan’s 2050 electricity consumption precisely—an essential consideration for energy policy and planning in the context of transitioning to nuclear and renewable energy.

Specifically we employ the following logarithmic version of multivariate autoregressive (AR(p)) model of log electricity consumption growth:


$$\text{ln}\left(EC_{t}\right)=\phi_{\text{0}}+\phi_{\text{1}}\text{ln}\left(\frac{EC_{t-\text{1}}}{EC_{t-\text{2}}}\right)+\sum_{j=\text{2}}^{p+\text{1}}\phi_{j}\text{ln}\left(EC_{t-j+\text{1}}\right)+\beta_{\text{1}}\text{ln}\left(Pop_{t-\text{1}}\right)+\beta_{\text{2}}\text{ln}\left(Age_{t-\text{1}}\right)+\beta_{\text{3}}\text{ln}\left(GDP_{t-\text{1}}\right)+\epsilon_{t}$$

where $EC_{t}$ represents the electricity power consumption in kilowatt-hour per capita in year t, $Pop_{t}\;$represents the population size of Japan in year t, $Age_{t}$ is the median age of Japanese (both sexes) for year t, ${GDP}_{t}$ is the GDP per capita of Japan denominated in US dollar for year t. We include five lag values of the dependent variable to control the autoregressive nature of electricity consumption. The multivariate AR(p) model with stationary regressor (change in electricity consumption from year t-2 to year t-1) has been chosen to model the autoregressive and cointegrating nature of empirical energy consumption [27,28]. See Sims, Stock and Watson [29] and Watson [30] for excellent exposition on modeling autoregressive processes. In addition to the two specifications above, we estimate a third specification in which the log population level is replaced by the lagged population growth rate; as explained in Section 4.2, this variant is used for the 2050 projection. Estimation results are reported in Section 4.2.

The specification can also be situated against the main alternatives used to project electricity demand, which speaks to its robustness. Relative to autoregressive distributed-lag and cointegration models of energy demand [27,28], our model is a restricted member of the same family: it retains the levels information exploited by cointegration methods. Section 4.3 verifies the cointegrating relationship directly. The relatively low number of parameters better suits a 44-year annual sample. Relative to structural optimization models of the kind used in supply-side studies of Japan [13–15], a reduced-form demand equation requires far fewer assumptions and exposes every driver to diagnostic testing, at the cost of abstracting from sectoral detail. Compare to machine-learning forecast methods, my model sacrifices flexibility for transparency and replicability: every coefficient is interpretable, and the diagnostic battery of Section 4.3—residual autocorrelation, unit roots, cointegration, autocorrelation-robust inference, omitted-variable and sample-horizon robustness—documents the respects in which the estimates are and are not fragile. This robustness-first design is why the projection is reported as a band across specifications, samples, and population paths rather than as a point forecast.

## 4. Empirical results

### 4.1. Descriptive statistics and correlations

Table 4 reports descriptive statistics for the estimation sample (1982–2021, N = 40) and Table 5 the corresponding Pearson correlation matrix. Two features stand out. First, log population is nearly constant within the sample: its standard deviation is 0.024 log points, against 0.187 for log electricity consumption and 0.446 for log GDP per capita. Japan’s population varied by barely ±2% around its mean over the four decades used for estimation, so any coefficient attached to the population level is identified from very limited variation—a point that becomes important for projection. Second, the log levels of consumption, population, GDP per capita, and median age are all highly correlated with one another (pairwise correlations of 0.85–0.99), as expected of trending macroeconomic series; only the lagged consumption growth rate is weakly correlated with the levels. These correlations motivate both the parsimonious specification and the cointegration analysis in Section 4.3.

**Table 4:** Descriptive statistics of the estimation sample (1982–2021, N = 40). EC is electricity consumption in kWh per capita; Pop is total population; GDP per capita is in current US dollars; Age is the median age of the population, interpolated between census years. Δln EC(t − 1) denotes ln[EC(t − 1)/EC(t − 2)].

Variable	Mean	Std. dev.	Min	Median	Max
ln EC (t)	8.887	0.187	8.403	8.975	9.080
Δln EC (t − 1)	0.014	0.033	−0.053	0.009	0.122
ln EC (t − 1)	8.872	0.201	8.398	8.972	9.080
ln Pop (t − 1)	18.646	0.024	18.583	18.657	18.668
ln GDP per capita (t − 1)	10.345	0.446	9.211	10.510	10.812
ln Age (t − 1)	3.716	0.108	3.498	3.730	3.854

**Table 5:** Pearson correlation matrix of the regression variables over the estimation sample (1982–2021).

	ln EC	Δln EC(−1)	ln EC(−1)	ln Pop(−1)	ln GDP(−1)	ln Age(−1)
ln EC	1.000	−0.305	0.988	0.983	0.957	0.881
Δln EC(−1)	−0.305	1.000	−0.312	−0.353	−0.289	−0.469
ln EC(−1)	0.988	−0.312	1.000	0.985	0.948	0.900
ln Pop(−1)	0.983	−0.353	0.985	1.000	0.933	0.907
ln GDP(−1)	0.957	−0.289	0.948	0.933	1.000	0.847
ln Age(−1)	0.881	−0.469	0.900	0.907	0.847	1.000

### 4.2. Regression results

Table 6 reports the regression results. Column (1) is the parsimonious specification with one lag of log consumption and the lagged consumption growth rate; column (2) adds four further lags of log consumption; column (3) replaces the log population level with the lagged population growth rate. Standard errors are heteroskedasticity-consistent (HC0) throughout.

**Table 6:** A log-growth model of electricity consumption, 1982–2021.

	(1)	(2)	(3) Projection
Dependent variable: ln EC (t)			
Δln EC (t − 1)	0.042	0.216**	−0.043
	(0.154)	(0.101)	(0.150)
ln EC (t − 1)	0.435***	0.282***	0.786***
	(0.147)	(0.090)	(0.083)
ln EC (t − 2)		0.066	
		(0.102)	
ln EC (t − 3)		0.248	
		(0.175)	
ln EC (t − 4)		−0.183	
		(0.213)	
ln EC (t − 5)		0.028	
		(0.099)	
ln Population (t − 1)	3.148***	3.101**	
	(0.974)	(1.325)	
Δln Population (t − 1)			−2.154
			(6.392)
ln GDP per capita (t − 1)	0.085***	0.093***	0.079**
	(0.028)	(0.027)	(0.031)
ln Median age (t − 1)	−0.123*	−0.126	−0.132
	(0.073)	(0.092)	(0.195)
Constant	−54.086***	−53.351**	1.588***
	(17.075)	(23.484)	(0.588)
F-statistic	444.60***	141.47***	348.29***
Adjusted R²	98.3%	96.9%	97.8%
Durbin–Watson	2.124	2.166	2.143
Breusch–Godfrey p (1 lag)	0.053	0.067	0.049
Breusch–Godfrey p (2 lags)	0.063	0.132	0.074
Estimation sample	1982–2021	1985–2021	1982–2021
Number of observations	40	37	40

The dependent variable is log electricity consumption per capita in year t. Columns (1) and (2) include the log population level; column (3), used for the 2050 projection in Section 4.4, replaces it with the lagged population growth rate. Heteroskedasticity-consistent (HC0) standard errors in parentheses. *, **, and *** denote significance at the 10%, 5%, and 1% levels. The estimation sample ends in 2021 because interpolated median age is available through the 2020 census; column (2) begins in 1985 because of the additional lags.

Three results in Table 6 matter for what follows. First, electricity consumption is persistent: the coefficient on lagged log consumption is 0.435 in column (1), implying a long-run multiplier of roughly 1.8 on permanent changes in the drivers. The additional lags in column (2) are individually and jointly small, so the parsimonious specification is retained as the baseline. Second, GDP per capita raises consumption—a short-run elasticity of 0.085 in column (1), or about 0.15 in the long run—while an older population lowers it, with a median-age coefficient of −0.123 (significant at the 10% level). Third, the population-level coefficient (3.148) is large and statistically significant but is identified from the very limited within-sample variation in log population documented in Table 4: the sample standard deviation of ln Pop is only 0.024, whereas Japan’s projected 2050 population lies more than seven sample standard deviations below the sample minimum. Extrapolating the level coefficient that far outside the estimation range is not defensible, and mechanical extrapolation drives projected consumption to implausibly low levels. For projection we therefore use column (3), which replaces the population level with the lagged population growth rate—the specification implicit in our description of the model as one of “change in population size.” In column (3) the persistence coefficient rises to 0.786 (long-run GDP-per-capita elasticity of about 0.37), and the population growth rate enters negatively but imprecisely; the level and growth-rate specifications are complementary views of the same cointegrated system, and Section 4.3 shows that the baseline results are robust across them.

### 4.3. Diagnostic tests and robustness

We first test for residual autocorrelation, as is appropriate for annual time series (the concern raised by both the Durbin–Watson and Breusch–Godfrey traditions). The Durbin–Watson statistics in Table 6 are close to 2 in all three columns (2.12–2.17), and the Breusch–Godfrey LM test is marginal at the first lag (p = 0.05–0.07) and insignificant at two lags for columns (1) and (2). As a corrective measure we re-estimated column (1) with Newey–West heteroskedasticity- and autocorrelation-consistent standard errors (two lags): all substantive conclusions survive, with the population level (p < 0.001), GDP per capita (p < 0.001), and median age (p = 0.046) all remaining significant. Reported inference is therefore not an artifact of serial correlation.

Second, we address the time-series properties of the data. Table 7 reports augmented Dickey–Fuller tests: none of the log-level series rejects a unit root, and only GDP per capita rejects in first differences, as expected in a sample of 44 annual observations where the power of unit-root tests is low. The appropriate question is therefore whether the levels regression is a cointegrating relationship. An Engle–Granger test on the residuals of the static levels regression rejects the null of no cointegration (t = −4.52, p = 0.015), supporting the interpretation of Table 6 as an error-correction-type representation of a cointegrated system, in the spirit of the autoregressive distributed-lag tradition in energy-demand modeling [27,28]. As Sims, Stock and Watson [29] show, coefficients on stationary combinations of cointegrated regressors retain standard asymptotic inference in levels autoregressions, which is the basis for the specification adopted here. We acknowledge that with 40 usable observations and up to nine estimated parameters in column (2), degrees of freedom are thin; this is precisely why the parsimonious column (1) is the baseline and why column (2) serves only to verify that the added lags change nothing of substance.

**Table 7:** Unit-root and cointegration tests. Augmented Dickey–Fuller statistics with MacKinnon p-values in parentheses; lag length selected by AIC (maximum 4). The Engle–Granger row tests the residuals of the static regression of ln EC on ln Population, ln GDP per capita, and ln Median age (each lagged one year) against the null of no cointegration.

Series	Level (constant + trend)	First difference (constant)
ln EC	−1.348 (0.876)	−1.925 (0.320)
ln Population	0.152 (0.995)	−0.755 (0.832)
ln GDP per capita	−1.334 (0.879)	−4.572 (<0.001)
ln Median age	2.151 (1.000)	1.424 (0.997)
Engle–Granger cointegration	t = −4.520 (p = 0.015)	

Fourth, we examine sensitivity to the sample horizon, which also answers the natural question of why the estimation sample stops before 2024–2025. The binding constraint is median age: it is observed only at quinquennial censuses and interpolated in between, so the interpolated series ends with the 2020 Census, and 2025 values of the other source series were not yet published when the analysis was conducted. Extending the sample to 2024 by continuing the census age trend linearly (thereby adding the declining-consumption years 2022–2024) leaves every conclusion intact: in the baseline specification the GDP-per-capita coefficient is essentially unchanged (0.083 versus 0.085), the aging effect remains negative, and the population and persistence terms retain their signs and significance (N = 43, 1982–2024). In the projection specification the steady state falls to about 8,570 kWh per capita, or roughly 900 TWh of total consumption—the origin of the lower edge of the projection band reported in Section 4.4. Because the extension rests on extrapolated rather than observed census data, the census-anchored 1982–2021 sample is retained as the baseline.

Third, Table 8 examines whether the baseline is vulnerable to omitted-variable bias from energy-sector and structural covariates, adding to column (1) of Table 6, one at a time: the lagged real energy price (the consumer energy price index deflated by the all-items CPI, OECD Main Economic Indicators); lagged urbanization (urban population share, World Bank); the lagged industry share of value added (World Bank, available from 1994); and the lagged nuclear and renewable shares of electricity generation (Ember/Energy Institute, available from 1985). The real energy price enters negatively and significantly (short-run elasticity −0.141, implying roughly −0.24 in the long run), consistent with standard energy-demand estimates, and its inclusion strengthens rather than weakens the baseline: the coefficients on population, GDP per capita, and lagged consumption retain their signs and significance, and the Breusch–Godfrey statistic becomes clearly insignificant (p = 0.51). Urbanization is insignificant—unsurprising, since Japan’s urban share trends almost linearly and is nearly collinear with the population and income variables. The industry share, available only from 1994, is insignificant on its shortened sample. Most importantly for the consistency of the study design, the generation-mix shares are individually insignificant: electricity demand does not depend on whether the electricity was produced by nuclear plants or renewables. Demand projection can therefore be conducted independently of the supply-mix scenarios analyzed in Section 5, and the parsimonious demographic-economic baseline is retained for projection. We do not carry the energy price into the 2050 projection because doing so would require an additional exogenous assumption about real energy prices three decades ahead; conditional on the other drivers, omitting a mean-reverting relative price affects the level of the projection only modestly.

**Table 8:** Robustness of the baseline specification to additional controls.

	(1) +Price	(2) +Urban	(3) +Industry	(4) +Mix
Δln EC (t − 1)	0.004	0.057	0.127	0.065
	(0.138)	(0.143)	(0.129)	(0.169)
ln EC (t − 1)	0.419***	0.404***	−0.081	0.444**
	(0.148)	(0.148)	(0.271)	(0.183)
ln Population (t − 1)	2.134**	3.137***	4.289**	5.833***
	(0.965)	(0.993)	(1.927)	(1.970)
ln GDP per capita (t − 1)	0.060**	0.080***	0.025	0.103***
	(0.025)	(0.027)	(0.046)	(0.028)
ln Median age (t − 1)	0.119	0.049	−0.113	−0.674
	(0.097)	(0.245)	(0.153)	(0.499)
ln Real energy price (t − 1)	−0.141***			
	(0.041)			
ln Urban share (t − 1)		−0.153		
		(0.205)		
ln Industry share (t − 1)			−0.120	
			(0.186)	
Nuclear share (t − 1)				−0.069
				(0.133)
Renewable share (t − 1)				0.770
				(0.503)
Constant	−35.694**	−53.515***	−69.744**	−102.461***
	(16.979)	(17.594)	(35.056)	(35.096)
Adjusted R²	98.4%	98.3%	65.4%	96.4%
Durbin–Watson	2.036	2.118	2.007	2.357
Breusch–Godfrey p (1 lag)	0.508	0.067	0.543	0.014
Estimation sample	1982–2021	1982–2021	1995–2021	1986–2021
Number of observations	40	40	27	36

Each column adds the indicated control(s) to column (1) of Table 6. Real energy price is the consumer energy price index deflated by the all-items CPI (OECD Main Economic Indicators, annual averages of monthly data); urban share and industry share of value added are from the World Bank World Development Indicators; nuclear and renewable shares of electricity generation are from Ember and the Energy Institute. HC0 standard errors in parentheses; *, **, *** denote significance at the 10%, 5%, and 1% levels. Samples differ because the industry share is available only from 1994 and the generation shares from 1985.

### 4.4. Projecting electricity consumption in 2050

To produce a projection of Japan’s electricity consumption in 2050, we combine the column (3) specification of Table 6 with external forecasts of the exogenous drivers, summarized in Table 9: a population change rate of −0.89% per year and a 2050 population of 105.1 million (World Bank population estimates and projections), a median age of 53.6 years (ESCAP), and GDP per capita of USD 64,809 (PwC). The fitted projection equation is:


$$\begin{array}{cc} \text{ln EC}(\text{t})\;= & \;\text{1}.\text{588 }-\;\text{0}.\text{043}\cdot \Delta \text{ln EC}(\text{t}-\text{1})\;+\;\text{0}.\text{786}\cdot \text{ln EC}(\text{t}-\text{1})\;-\;\text{2}.\text{154}\cdot \Delta \text{ln Pop}(\text{t}-\text{1})\;+\\ & \;\text{0}.\text{079}\cdot \text{ln GDP}(\text{t}-\text{1})\;-\;\text{0}.\text{132}\cdot \text{ln Age}(\text{t}-\text{1}) \end{array}$$

**Table 9:** Projected values of the exogenous variables for 2050.

Variables	Forecast data	Period	Source
population	− 0.89%	2050	World Bank Database
median age	53.6	2050	ESCAP
GDP per capita	USD 64,809	2050	PwC

Because the equation is dynamic, we evaluate it at its steady state, in which the growth of consumption has levelled off (Δln EC = 0) and consumption is constant at EC*. Setting ln EC(t) = ln EC(t − 1) = ln EC* and solving gives ln EC* = [1.588 − 2.154 × (−0.0089) + 0.079 × ln(64,809) − 0.132 × ln(53.6)]/ (1 − 0.786), which yields EC* ≈ 9,514 kWh per capita. Multiplying by the projected 2050 population of 105.1 million gives total consumption of approximately 999 TWh—roughly 1,000 TWh. The projection is robust in level terms across reasonable variations: using the extended-lag analogue of the projection specification gives 9,176 kWh per capita and 964 TWh, and compounding the population forward at −0.89% per year instead of using the World Bank 2050 level gives 935 TWh. We therefore work with a projection band of roughly 935–1,000 TWh—which widens to roughly 900–1,000 TWh under the extended-sample variant of Section 4.3—and adopt 999 TWh as the planning benchmark.

The economics of the projection deserve to be spelled out, because declining population and rising GDP per capita pull in opposite directions and their netting drives every downstream quantity in this paper. On a per-capita basis, consumption rises from 7,655 kWh in 2023 to a projected 9,514 kWh in 2050, an increase of about 24%. Decomposing this change using the long-run coefficients of the projection specification: the near-doubling of GDP per capita (USD 35,215 to USD 64,809) contributes approximately +25%; the rise in median age from 47.0 to 53.6 years subtracts about 8%; the slightly faster population decline adds roughly 4% (population shrinkage raises per-capita consumption marginally, for example through smaller households and fixed infrastructure loads); and convergence from the below-steady-state 2023 level accounts for the remainder. In total terms, per-capita consumption rising 24% while population falls 16% (124.5 to 105.1 million) yields total consumption about 5% above the 2023 level of 953 TWh. Two conventions should be noted. First, the model is estimated on consumption per capita (the World Bank definition), so the projection is of final consumption; since transmission and distribution losses mean required generation exceeds final consumption, comparing projected consumption with generation capability in Section 5 is conservative. Second, all monetary inputs are in current US dollars, matching the estimation data. With this projection in hand, we can now design and discuss the use of nuclear and renewable energy to replace fossil-fuel generation.

## 5. Potential replacement of fossil fuel: Nuclear and renewables

### 5.1. Nuclear potentials

One of the principal elements of Japan’s nuclear strategy is restarting existing reactors and constructing new ones. Prior to the Fukushima accident, Japan had 54 operable nuclear reactors at 16 sites; in the wake of the accident, the government shut down all of them, as safety concerns fueled widespread public opposition to nuclear energy. Since then, Japan’s nuclear fleet has undergone significant scrutiny and transformation. Over the following years, only a limited number of reactors were restarted, owing to stricter safety requirements and continuing public concern: by the end of 2023, only 12 of Japan’s 33 operable reactors had been restarted [31], representing 21.4% of total nuclear capacity, or 75.7 TWh of electricity generation. Details of the status of Japan’s operable nuclear power plants are provided in S1 Table, and the three units currently under construction are listed in S2 Table. Because the government aims for nuclear power to supply approximately 20–22% of electricity by 2030, achieving this will require restarting more reactors and possibly building new facilities.

Since the end of 2023, the pace of restarts has quickened. Two boiling-water reactors—Onagawa Unit 2 (October 2024) and Shimane Unit 2 (December 2024)—returned to service, the first reactors of the same type as those at Fukushima Daiichi to be restarted, and in January 2026 the Tokyo Electric Power Company restarted Unit 6 of the Kashiwazaki-Kariwa station, the world’s largest nuclear plant and the company’s first reactor to resume operation since 2011 [32]. With these additions, 15 of Japan’s 33 operable reactors are now operating or cleared to operate, lifting nuclear’s share of electricity generation from about 7.7% in 2023 toward roughly 9% in 2024 and pushing annual nuclear output to its highest level since the 2011 accident [33]. The remaining eighteen units stay offline pending safety screening and local consent, however, and no further restarts are scheduled for fiscal 2026, which reinforces the central argument of this paper that public trust and regulatory clearance, rather than engineering capacity, set the pace at which nuclear power can return to Japan’s energy mix. Fig 1 contrasts the status of the fleet recorded in this study (April 2024) with the most recent picture (2026), making visible both the progress achieved and the capacity that remains idle.

**Fig 1:**
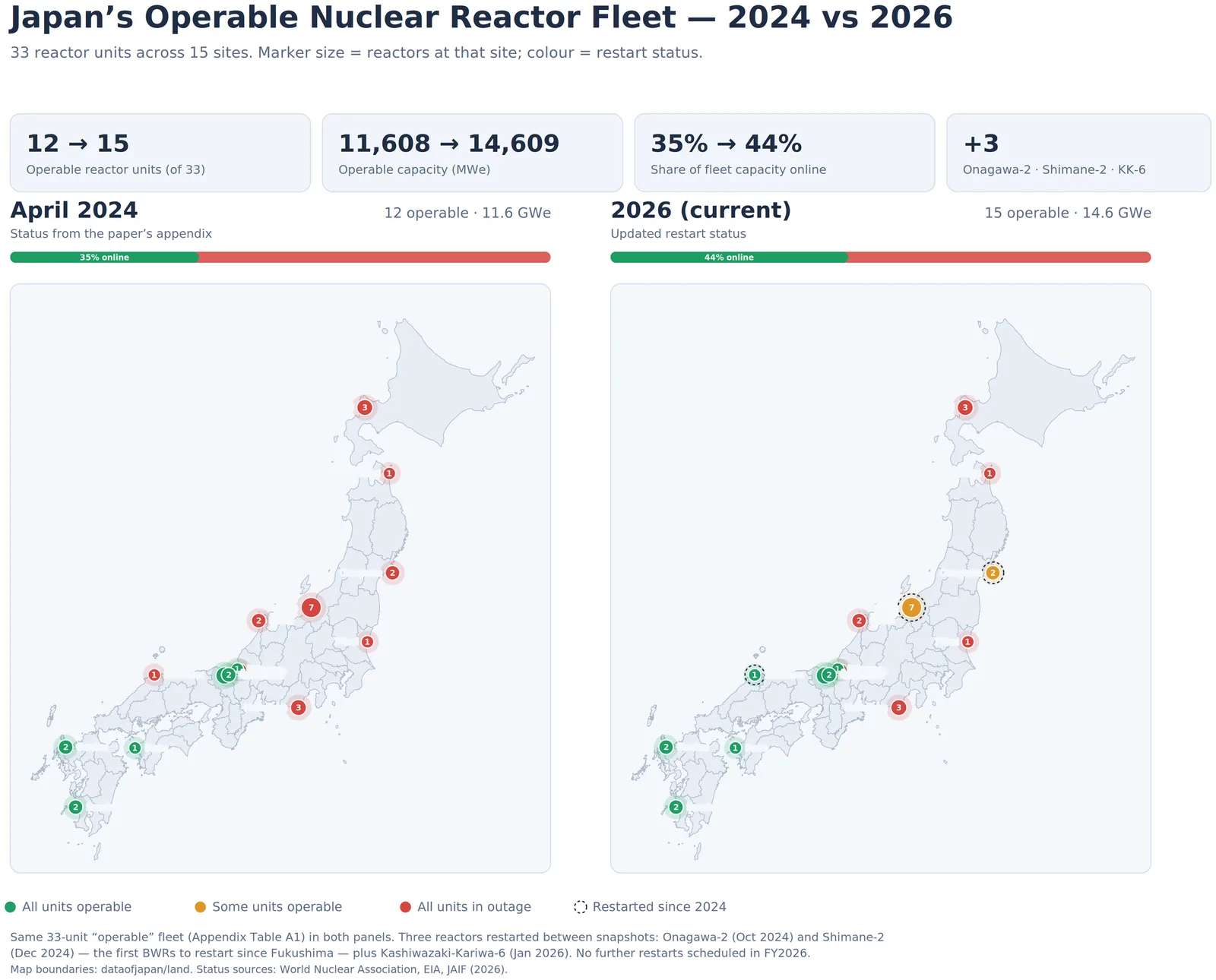
Status of Japan’s operable nuclear reactor fleet, April 2024 versus 2026. Basemap drawn by the author from dataofjapan/land (a conversion of Global Map Japan, Geospatial Information Authority of Japan), used and adapted under the GSI Content Terms of Use (Public Data License v1.0, compatible with CC BY 4.0).

As noted above, nuclear energy provides a steady, reliable supply that can offset the intermittency of renewable sources, supporting a diversified and balanced energy system in Japan. Generation IV reactors, such as the Supercritical-Water-Cooled Reactor (SCWR), promise even greater efficiency, sustainability, and safety. These advanced reactors are designed to use fuel more efficiently, generate less waste, and provide inherent safety features that reduce the chance of serious accidents [34]. Investing in such cutting-edge technologies could position Japan as a leader in next-generation nuclear power.

Public perception of nuclear energy remains mixed and poses a major challenge for Japan. The legacy of the Fukushima disaster has produced widespread skepticism and opposition, prompting protests and calls for greater investment in renewable energy. Public fears about safety, environmental contamination from nuclear leaks, and the long-term management of nuclear waste are significant barriers the sector must overcome [35]. Rebuilding public trust will require transparent communication about safety protocols, emergency-preparedness plans, and regulatory oversight. Proactively engaging communities in discussion of the benefits and risks of nuclear energy will be essential; through open dialogue and education, the sector can help ease fears and build confidence among stakeholders. Moreover, because nuclear energy generates electricity with minimal greenhouse gas emissions, demonstrating its role in achieving broader environmental goals can further strengthen social acceptance.

Beyond its environmental benefits, integrating nuclear power into Japan’s broader energy strategy could also advance the country’s energy-independence goals [36]. As noted earlier, Japan has historically relied on imported fossil fuels, leaving it vulnerable to external disruption and price fluctuations. By investing in flexible energy resources—particularly nuclear power combined with renewables—Japan can reduce its reliance on foreign supply while enhancing energy security. A diversified mix that includes a significant share of nuclear power could provide a more stable, reliable system and help insulate the nation from global market volatility.

Overall, there is an urgent need to restart existing reactors and develop new plants to support a sustainable energy future; doing so would also help Japan build a more resilient energy system and strengthen its energy security.

### 5.2. Renewable energy potentials

The transition to renewable energy is equally essential under the Paris Agreement. Moreover, investing in renewable energy technologies can promote economic growth, create jobs, and spur technological innovation [37].

Japan has diverse and considerable renewable resources, but solar energy stands out as one of the best options, thanks to its geographic suitability, technological progress, favorable economics, and ease of integration with existing infrastructure. Japanese scholars have recently argued that solar energy has great potential for the country’s energy transition [38].

Japan’s geography makes it well suited to solar power. The country receives ample sunlight throughout the year, especially in regions such as Hokkaido, Kyushu, Shikoku, and Kanto, where it is abundant and intense enough to support extensive solar photovoltaic (PV) installations [39]. Its varied geography and range of climates, combined with a dense population and high degree of urbanization, make the Japanese archipelago a favorable environment for solar installations both on rooftops and at ground level. Solar panels also offer more flexibility in siting than wind energy, which is generally limited to areas with large open spaces or coastal access; they can be deployed on many surfaces, from residential rooftops to commercial buildings. This flexibility allows solar PV to integrate seamlessly into existing urban and rural infrastructure while avoiding large-scale land modification [40].

Technological progress has substantially improved the efficiency and reduced the cost of solar energy, making it competitive with other renewable sources. Continued development of PV technologies—such as monocrystalline and polycrystalline silicon panels, thin-film technologies, and bifacial modules—has significantly improved energy-conversion efficiency. Advanced panels can now convert more than 20% of captured sunlight into electricity, and ongoing research points to even higher efficiencies in the coming years [41]. Improvements in manufacturing have also driven costs down dramatically: over the past decade, the cost of solar PV electricity has fallen by more than 90%, making it more attractive than traditional fossil fuels and other renewables, which typically require larger upfront investment and longer payback periods [39].

Solar energy is widely regarded as a financially viable way to meet Japan’s energy needs while creating jobs and attracting investment. The Feed-in Tariff (FiT) scheme has greatly benefited the sector: by guaranteeing fixed prices for solar-generated power, it reduces financial risk for investors and has driven rapid expansion of solar projects nationwide. The favorable investment environment it created has attracted both domestic and foreign investors, fueling considerable industry growth. As a result, Japan has become one of the world’s largest solar PV markets, and employment in the sector has expanded across functions ranging from manufacturing and installation to maintenance and after-sales service [37].

Japan’s location and economy also make it well suited to wind energy, though its advantages are not as great as those of solar PV. The country’s long coastline and access to strong, steady winds create a favorable environment for wind power. Several studies estimate Japan’s wind energy potential at between 296 GW and 938 GW [15]. Wind farms can be built near major cities and coastal industrial areas, enabling efficient transmission with lower losses. Technological progress is especially valuable here: because Japan’s coastal waters are deep and less suitable for conventional turbines, recent breakthroughs allow wind farms to be installed farther offshore in deeper water, tapping more powerful winds while reducing ecological impact.

Because Japan lies along the Pacific “Ring of Fire” and has significant geothermal activity, geothermal energy is often seen as another promising renewable resource, even though its development has been relatively slow. Exploiting geothermal resources typically involves high upfront costs for drilling and exploration, which carry the inherent risk of not finding a viable reservoir. Geothermal projects also often face opposition from local communities concerned about effects on hot springs, which are culturally and economically important in Japan. These factors, together with the long lead times required to develop geothermal plants, limit the scalability and appeal of geothermal energy relative to the more rapidly deployable solar PV [42].

Hydropower has long been an important part of Japan’s renewable energy portfolio. However, the potential for new large-scale hydro projects is constrained by geographical limits and environmental concerns. Most suitable sites for large dams have already been developed, and further expansion could cause ecological disruption and displacement. Conventional hydropower in Japan is therefore considered nearly fully developed, with little room to add capacity. Some potential remains for small and micro-hydropower, especially in mountainous regions, but its contribution to the national grid is minimal compared with large-scale solar, and strong seasonal fluctuation in output is a further limitation [43].

On balance, solar energy offers the greatest benefits, given its declining costs, ease of installation, and scalability. Japan’s well-established grid also supports widespread solar adoption: it can connect and integrate solar panels distributed across the country, while smart-grid and energy-storage technologies have mitigated problems of intermittency and variability, enhancing the reliability and stability of solar power.

Public acceptance and support for solar energy in Japan are also generally high, owing to its cleanliness, which further encourages adoption [44]. Permits are relatively easy to obtain and opposition is minimal compared with wind and other renewables, streamlining deployment and lowering regulatory and social barriers.

In sum, although Japan’s renewable resources are diverse, solar and wind power stand out as the most promising options because of their geographical suitability, technological maturity, economic feasibility, and ease of integration with existing infrastructure. While geothermal and hydropower also contribute to Japan’s current energy mix, the advantages of solar PV and wind turbines position them as the leading drivers of the energy transition.

### 5.3. Bridging the gap by nuclear and renewable energy in different scenarios

Japan currently has 33 operable nuclear reactors with a combined output capability of 277.50 TWh [31]. Assuming all of them are restarted by 2050, nuclear power could form a significant part of the country’s energy mix. Even so, approximately 469 TWh of additional clean energy would still be needed for a complete shift away from fossil fuels (Table 10). Closing this 469 TWh gap would require a substantial increase in nuclear capacity, achievable through strategic initiatives such as building latest-generation reactors and improving the efficiency of existing ones.

**Table 10:** Clean energy gap by 2050.

2050 Electricity Consumption(Projected)		Current nuclear power capability		Current renewable energy capability		Clean energy gap
999 TWh	-	277.5 TWh	-	252.75 TWh	=	469 TWh

### 5.4. How many new reactors need to be constructed?

Japan would need to expand its nuclear fleet substantially: with the existing 33 reactors generating an average of 8.41 TWh each [31], closing the 469 TWh gap would require building approximately 56 additional reactors (Table 11). This calculation assumes the current scale and efficiency of Japan’s operational reactors are maintained.

**Table 11:** New reactors to be constructed to bridge the energy gap.

Operable reactors	Nuclear power capability	Average capability per unit	Energy gap	New reactors to be constructed
33	277.50 TWh	8.41 TWh	469 TWh	55.77

The 8.41 TWh average output capability per unit is total operable capability (277.5 TWh) [31] divided by 33 units; Section 5.3 discusses the capacity-factor sensitivity of this figure.

Two qualifications attach to this arithmetic. The first concerns the 8.41 TWh figure itself. It is the average annual output capability per operable unit—total capability of 277.5 TWh [31] divided by 33 units—which corresponds to operating the 33.1 GWe fleet at a capacity factor of roughly 96%. It is therefore a capability ceiling rather than a forecast of realized output. Japan’s post-Fukushima restart experience has delivered fleet capacity factors well below the world average of about 83% [10], reflecting staged restarts, extended safety outages, and periodic judicial injunctions. If new reactors were instead to operate at an 80% capacity factor, the average unit would deliver about 7.0 TWh per year and the requirement would rise to roughly 67 reactors; at 70%, to about 76. The reactor counts in Table 11 should accordingly be read as a lower bound, with 56–67 units the policy-relevant range under realistic operating assumptions.

The second qualification concerns timing. Nuclear construction programs typically require ten to twenty years per plant from siting decision to commercial operation once licensing, public consultation, and construction are included. Japanese vendors have historically built advanced boiling-water reactors in as little as four to five years of physical construction (Kashiwazaki-Kariwa Units 6 and 7), but post-Fukushima licensing and local consent, not construction engineering, are now the pacing items. Completing 56–67 units by 2050 would therefore require siting and licensing to begin essentially immediately and to proceed in parallel across many sites, together with sustained reinvestment in the certified nuclear supply chain (heavy forgings, containment fabrication) and in a specialized workforce that has contracted since 2011. We accordingly characterize the all-nuclear pathway as conditionally feasible: achievable on engineering grounds only if licensing throughput and industrial capacity are scaled up during the 2020s, and otherwise an argument for the mixed nuclear-renewable scenario below, which nearly halves the required number of new units.

Tripling the reactor fleet, however, is not only an engineering challenge. For many residents the memory of the 2011 Fukushima Daiichi disaster remains vivid, and an all-nuclear expansion of this scale—a new plant in roughly half of Japan’s coastal prefectures—may be emotionally unacceptable regardless of its technical safety. This motivates a second pathway in which nuclear power shares the burden with renewable energy, led by solar. An alternative scenario assumes that Japan replaces its current fossil-fuel generation using nuclear power and renewable energy in equal shares, each supplying 50% of the gap (Table 12). Under this scenario, the nuclear requirement would be 234.5 TWh, necessitating approximately 28 new reactors, while the renewable component would require an additional 234.5 TWh of generation.

**Table 12:** Different scenarios to bridge the energy gap.

Scenarios	Nuclear Power gap	Renewable Energy gap
All fuel fossil energy replace by nuclear power	469 TWh	0TWh
50% Nuclear + 50% Renewable power	234.5 TWh	234.5 TWh

Achieving this would also require substantial investment in renewable technologies: the remaining gap is equivalent to roughly 2.1 times Japan’s current installed solar photovoltaic (PV) capacity [22]. The appeal of this mixed pathway is as much social as technical. Halving the nuclear requirement—from about 56 new reactors to roughly 28—proportionally reduces the number of new plants, emergency planning zones, and host communities whose consent must be won, while the balance is carried largely by solar power, which enjoys high public acceptance [44] and faces the fewest siting constraints among Japan’s renewables (Section 5.2). For a public whose trust was broken at Fukushima, a transition in which nuclear power leads but does not monopolize the energy mix may therefore be the more credible route to 2050; the siting framework developed in Section 5.4 applies with equal force under this scenario, but to a smaller and more easily accommodated fleet.

### 5.5. Where to build: Siting new reactors to minimize societal risk

Determining how many reactors to build settles only part of the expansion question; the other part is where to build them. The dominant societal cost of nuclear power is the risk that a severe accident poses to nearby residents, which regulators formalize through emergency planning zones (EPZs)—areas around a plant for which evacuation, sheltering, and food-control measures must be pre-arranged. The United States defines a 50-mile (about 80 km) ingestion-pathway EPZ, and we adopt this conservative radius as a measure of the population a plant could place at risk [45]. The all-nuclear scenario implies approximately 56 new reactors on a capability basis, or up to 67 once realistic capacity factors are applied (Section 5.3); grouping the upper end of this range into plants of three units each implies about 21 new plant sites, which we adopt as the planning benchmark for the siting analysis. Because every nuclear plant in Japan is sited on the coast for cooling water, we ask a concrete spatial question: where along Japan’s coastline should these 21 plants be placed so that the population inside their EPZs, and hence the potential human impact of an accident, is as small as possible?

Placing the 21 plants at random along the coast illustrates the stakes. Such a configuration draws roughly half of each EPZ over the sea, yet still encloses about 93 million residents (the median of 500 random configurations), about two-thirds of the population captured in our city dataset, because random coasts frequently fall near major metropolitan areas (Fig 2a). Deliberately steering the plants toward the least-populated coastlines instead, while keeping them spaced apart, lowers the enclosed population dramatically, to roughly 0.6 million (Fig 2b). The two maps make the difference visible: the optimized zones hug the empty coasts of Hokkaido, the San’in region, and remote stretches of Kyushu and Shikoku, avoiding the dense Tokyo-Osaka-Nagoya corridor.

**Fig 2:**
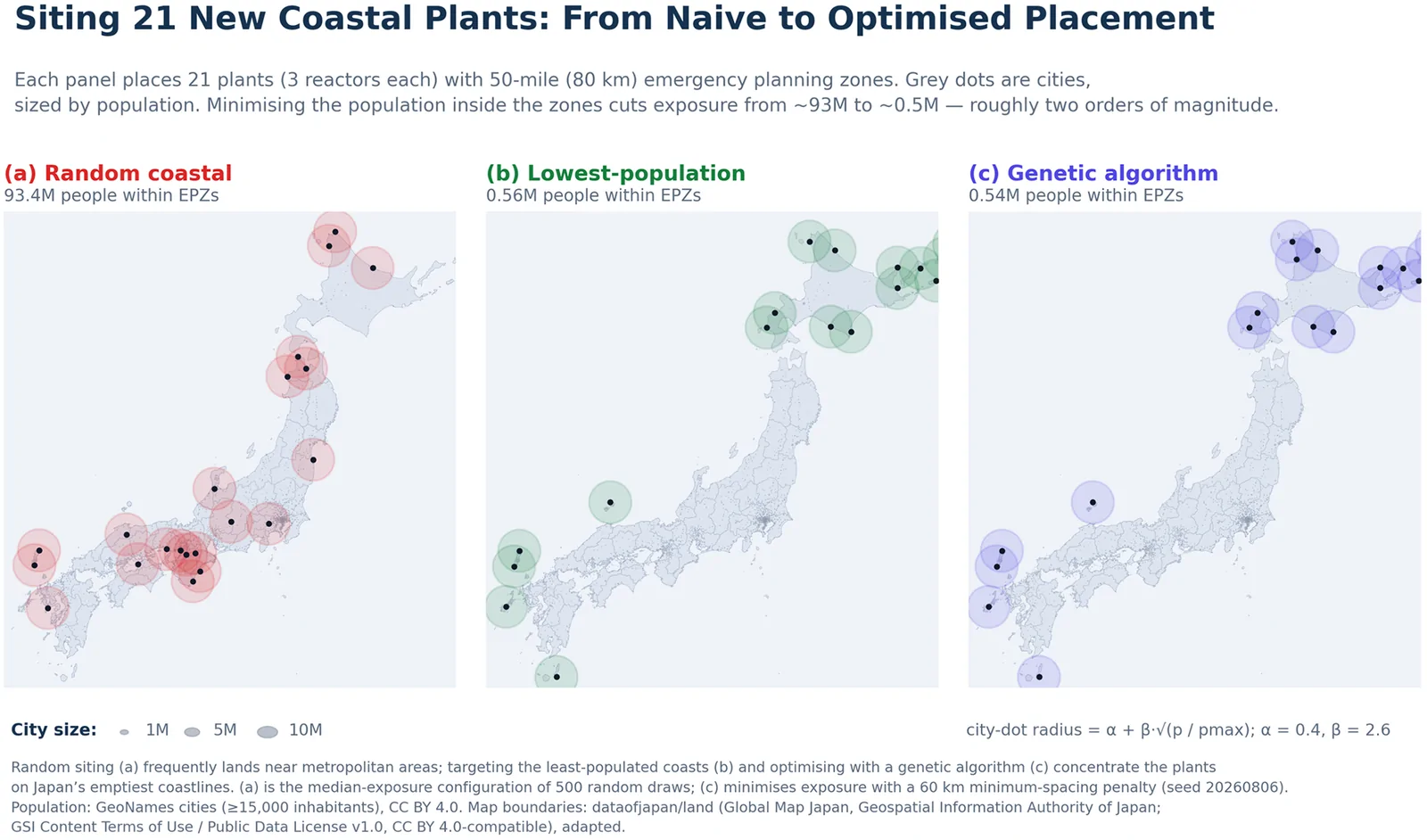
Three strategies for siting 21 new plants: (a) random coastal placement, (b) lowest-population siting, and (c) genetic-algorithm optimization of the exposed population. City symbols are drawn with radius α + β√(p/p_max_), where p is city population and p_max_ that of the largest city (Tokyo); α = 0.4 sets a minimum visible size and β = 2.6 the scale, so symbol area is approximately proportional to population. Map boundaries: dataofjapan/land (Global Map Japan, Geospatial Information Authority of Japan; GSI Content Terms of Use/ Public Data License v1.0, CC BY 4.0-compatible), adapted. Population data: GeoNames cities of at least 15,000 inhabitants (CC BY 4.0). Configurations shown are the reproducible re-run in the analysis package: panel (a) is the median-exposure configuration of 500 random draws and panel (c) applies a 60 km minimum-spacing penalty (random seed 20260806).

Formally, this is an obnoxious (undesirable) facility-location problem: choosing sites to minimize, rather than maximise, the population served. Because the objective is non-convex and combinatorial, EPZs overlap and sites are confined to the coastline, we solve it with metaheuristic optimization from the machine-learning family rather than with closed-form methods. We first applied a greedy heuristic and then a genetic algorithm (GA), in which candidate sets of 21 sites are encoded as individuals, recombined, and mutated over successive generations to minimize the union population inside the EPZs together with a dispersion term that discourages clustering. The GA converged to about 0.54 million people exposed (Fig 2c), slightly below the greedy solution and more than one hundred times smaller than the random benchmark, showing that exposure can be reduced by roughly two orders of magnitude through siting alone.

Minimising exposure in isolation, however, ignores that electricity must reach the population that consumes it: pushing plants toward empty coasts lengthens the distance over which power must be transmitted. We therefore treat siting as a two-objective problem and solve it with NSGA-II, an elitist multi-objective genetic algorithm that returns a Pareto front of non-dominated designs [46]. The first objective is the EPZ population (emergency exposure); the second is the annual transmission-loss cost of delivering power to that population, obtained by valuing the energy lost over the population-weighted delivery distance (530 TWh per year of generation, a loss rate of 4.5% per 1,000 km, and an electricity price of US$0.12 per kWh). The resulting front spans from a grid-first design exposing about 117 million people at only about US$122 million per year, to a safety-first design exposing about 0.4 million at about US$1,140 million per year (Fig 3). The knee of the front, the point of best compromise, exposes about 12 million people at about US$316 million per year.

**Fig 3:**
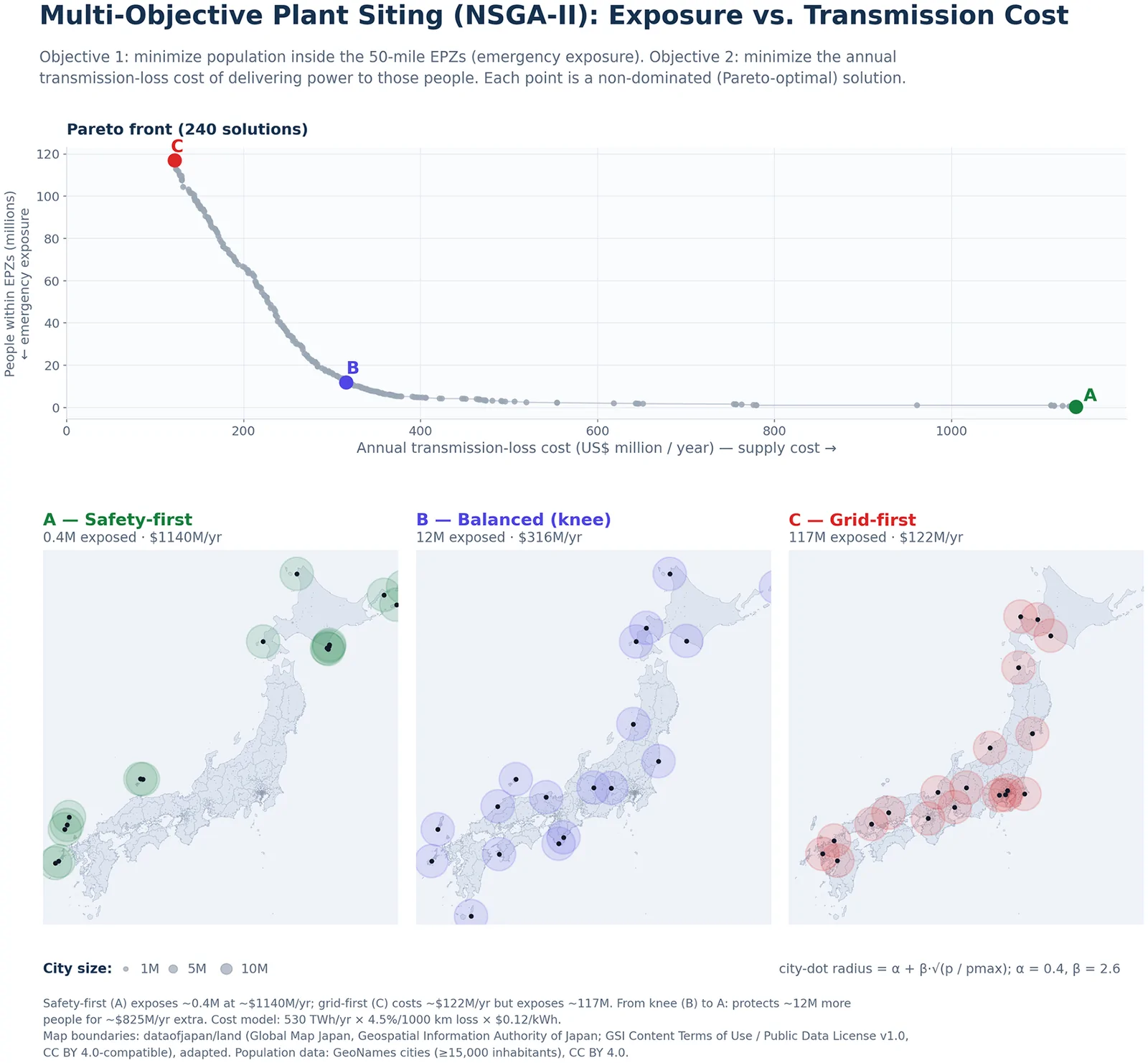
Multi-objective (NSGA-II) siting: emergency exposure versus transmission cost. City symbols are scaled as in Fig 2. Map boundaries: dataofjapan/land (Global Map Japan, Geospatial Information Authority of Japan; GSI Content Terms of Use/ Public Data License v1.0, CC BY 4.0-compatible), adapted. Population data: GeoNames cities of at least 15,000 inhabitants (CC BY 4.0). The 240-solution front shown is the reproducible re-run included in the analysis package (random seed 20260802).

The Pareto front quantifies the trade-off at the heart of public opposition to nuclear power. Moving from the balanced knee design to the safety-first design protects roughly 11.6 million additional people, but more than triples the population-weighted delivery distance and raises the annual transmission-loss cost by about US$824 million (on the order of US$15 billion in present value). The knee solution is therefore the most defensible single recommendation: it captures most of the achievable reduction in human exposure before supply costs rise steeply. These estimates are illustrative rather than definitive: population is measured from cities of at least 15,000 inhabitants [47], so absolute exposure is understated; the transmission cost is a simplified proxy; and a full siting study would add seismic hazard, coastline suitability, grid topology, and local consent as further constraints. Nonetheless, the analysis shows that the societal risk emphasised throughout this paper is not fixed but is itself an object of design: with appropriate optimization, Japan could expand nuclear capacity while holding the population exposed to a potential emergency to a small fraction of the naive baseline.

The omission of seismic and tsunami hazard from the optimization deserves emphasis rather than a passing caveat, because ground shaking and tsunami inundation—not population proximity—were the physical failure modes at Fukushima Daiichi. Minimizing the population inside emergency planning zones reduces the consequence term of societal risk but leaves the probability term unaddressed, and a remote coastline is not necessarily a low-hazard coastline: parts of the sparsely populated Pacific coasts selected by the optimizer lie near subduction-zone segments capable of generating large tsunamis. Two points follow. First, in Japanese practice site-level hazard is governed by the Nuclear Regulation Authority’s screening, which any candidate site must pass before considerations of population exposure become relevant; our framework should therefore be read as optimizing among hazard-cleared sites, not as a substitute for hazard assessment. Second, the methodology extends directly: seismic and tsunami hazard layers can be incorporated either as hard constraints—excluding coastline segments above a peak-ground-acceleration or tsunami run-up threshold—or as a third objective in the NSGA-II formulation, without changing the architecture of the analysis. The policy principle is that reduced population exposure must not be purchased with increased accident probability: the two dimensions of risk are complements in rebuilding public trust, not substitutes.

## 6. Discussion

Our demand estimates sit comfortably within the energy-demand literature from which the specification descends. The long-run income elasticity implied by the baseline (roughly 0.15, rising to about 0.37 in the projection specification) is below unity, consistent with the mature-economy estimates surveyed in the autoregressive distributed-lag tradition [27,28], and the short-run real energy price elasticity of −0.141 estimated in the robustness analysis (long-run roughly −0.24) falls within the conventional range for aggregate electricity demand. Where Japan differs from the economies studied in that earlier literature is in the strength of the demand dampeners: decades of aggressive efficiency policy—most prominently the Top Runner program [25]—, appliance saturation, and the offshoring of energy-intensive manufacturing plausibly explain why measured income elasticities are at the low end of international estimates.

The demographic results carry an implication that distinguishes Japan from most economies for which electricity demand has been modeled: aging measurably restrains electricity demand, with a median-age coefficient of −0.123 in the baseline. This is consistent with the mechanism discussed in Section 3.1—an older population reduces aggregate economic activity even as residential use per household may rise—and it partially offsets income-driven growth in the projection. The net outcome, per-capita consumption rising while total consumption grows only modestly, is the quantitative signature of an aging, shrinking, but still-enriching economy.

Beyond Japan, the demand-side determinants examined here connect to a broader recent literature on the drivers of sustainable energy transitions. Energy efficiency, human development, and renewable deployment jointly improve environmental carrying capacity as measured by the load capacity factor [48]; renewable-energy deployment supports sustainable development well beyond the high-income setting studied here [49]; and artificial-intelligence technologies are reshaping energy security through global supply chains [50]. These channels complement the demographic and income drivers modeled in this paper and bear directly on how efficiently a given demand projection can be met.

Our projection of approximately 1,000 TWh of consumption in 2050 provides a demand-side anchor for the supply-side literature on Japan’s carbon-neutral energy mix. Studies in that literature—cost-minimizing mix optimizations under siting constraints [13], marginal-cost analyses of fully renewable supply [14], and 100% renewable feasibility studies [15]—typically treat 2050 demand as a scenario input rather than an estimated quantity. Our contribution is complementary: an econometrically estimated, diagnostically tested demand benchmark with an explicit uncertainty band (935–1,000 TWh), which such supply-side studies can adopt directly. The finding that generation-mix shares do not enter the demand equation (Table 8) supports precisely this division of labor: demand can be projected independently of how the electricity is produced, and the mix can then be optimized conditional on demand.

The comparison with official policy is also instructive. The government’s Strategic Energy Plan targets a 2030 electricity mix of 20–22% nuclear and 36–38% renewables [6]. Our all-nuclear scenario is an analytical upper bound rather than a forecast, and the 50–50 scenario of Section 5.3—about 28 new reactors plus 234.5 TWh of additional renewable generation—is the pathway more nearly aligned with the policy trajectory. Viewed against Kharecha and Sato’s [19] accounting of the emissions cost of the post-Fukushima nuclear suspension, both pathways underline the same conclusion: the pace at which public trust allows nuclear capacity to return and expand, rather than any engineering parameter, is the state variable that determines whether the 2050 target is reachable.

Several limitations bound the analysis and mark out further research. On the demand side, the model is national and aggregate: it abstracts from sectoral and prefectural detail, from structural demand shifts such as transport electrification and data-center growth, and from relative prices in the projection itself (prices enter only the robustness analysis, because projecting them to 2050 would require another exogenous assumption); using observations after 2021 requires extrapolating median age beyond the 2020 Census (Section 4.3). On the supply side, the reactor arithmetic treats unit capability and capacity factors as policy-invariant, hydrogen is excluded on the grounds documented in Section 2.1, and the siting optimization omits seismic and tsunami hazard layers, land acquisition, and grid topology (Section 5.4). Further research could disaggregate demand by sector and region; endogenize efficiency policy, whose leverage is quantified in Section 7; incorporate hazard layers and grid constraints as additional objectives in the evolutionary siting framework; and re-estimate the model as the 2025 Census and post-2024 consumption data are published. The code and data released with this paper are designed to make such extensions straightforward.

## 7. Conclusion

This paper set out to quantify what Japan’s pledge of carbon neutrality by 2050 requires of its electricity sector. The empirical analysis yields four specific findings. First, electricity consumption over 1980–2023 is well described by a cointegrated autoregressive system in which income raises demand (long-run elasticity of roughly 0.15–0.37 across specifications), aging restrains it (median-age coefficient of −0.123), and real energy prices reduce it (short-run elasticity of −0.141); these conclusions are robust to autocorrelation-consistent inference and to the inclusion of urbanization, industrial structure, and generation-mix controls. Second, evaluating the projection specification at external 2050 forecasts of its drivers yields consumption of approximately 999 TWh (band: 900–1,000 TWh), about 5% above the 2023 level—rising per-capita demand nearly offset by a shrinking population. Third, after netting out the 530 TWh that existing nuclear and renewable capability could supply, a clean-energy gap of roughly 469 TWh remains, equivalent to about 56 new reactors at current average unit capability and up to 67 at the capacity factors Japan has realized since Fukushima; a 50–50 nuclear-renewable split halves the nuclear requirement to about 28 units. Fourth, siting is a design variable of first-order importance: optimized coastal siting reduces the population inside emergency planning zones by roughly two orders of magnitude relative to naive placement, at a transmission-loss cost of a few hundred million US dollars per year.

Finally, the siting analysis yields a policy lesson that goes beyond the arithmetic of capacity: where the new reactors are built matters as much as how many are built. Placing the required 21 plants at random along Japan’s coast would put roughly 93 million people inside a 50-mile emergency planning zone, whereas optimized siting on the least-populated coastlines reduces this figure to below one million (Section 5.4). The multi-objective analysis shows that this protection is inexpensive: relative to a grid-oriented configuration, a safety-first configuration raises annual transmission-loss costs by roughly US$0.2 billion per year at the knee-point compromise, and by about US$1 billion at the safety-first extreme—still orders of magnitude below the economic and social damage of a severe accident near a population centre. We therefore conclude that siting policy should take safety over efficiency. Deliberately accepting a modest and quantifiable supply cost in exchange for a reduction of roughly two orders of magnitude in the exposed population is sound risk management, and it is also the most credible way to rebuild the public trust that this paper identifies as the binding constraint on Japan’s nuclear future.

These findings support specific policy recommendations. (1) Because licensing throughput, not construction engineering, paces nuclear expansion, regulatory and consent processes for restarts and new builds should be resourced and scheduled as the program’s critical path, beginning in the 2020s. (2) New-build planning should use the 56–67 reactor range—not the capability-basis lower bound—and treat realized capacity factors as a policy variable worth improving. (3) Siting policy should minimize emergency-planning-zone population subject to the Nuclear Regulation Authority’s seismic and tsunami screening, in the spirit of the knee-point compromise identified by the multi-objective analysis, and communicate that trade-off transparently to host communities. (4) The mixed nuclear-renewable pathway—about 28 reactors plus roughly 234.5 TWh of additional renewable generation, led by solar—should be developed in parallel as the socially robust alternative, since it nearly halves the number of host communities whose consent must be won. (5) Demand-side efficiency remains quantitatively powerful: each 1% of avoided 2050 consumption removes the equivalent of about 1.2 reactors from the required build-out. Coordinated action on these fronts—with the rebuilding of public trust as the through-line—is, on our estimates, the difference between a reachable 2050 target and an aspirational one.

## Supporting information

S1 Code SI code data. (ZIP)

S1 Table In operation and operable nuclear reactors of Japan. Owner, plant, reactor type, output (MWe), year of commercial operation, age, and current status of the 33 operable units. Updated as of April 2024. Source: World Nuclear Association.
(DOCX)

S2 Table Nuclear reactors under construction in Japan. The three units under construction, with the same fields. Updated as of April 2024. Source: World Nuclear Association.
(DOCX)
