# Supplementary material for: Transition from fossil fuels to nuclear and renewable energy: Evidence from Japan
Source: PLoS One. 2026 Nov 13;21(11):e0353734. doi: 10.1371/journal.pone.0353734 (PMC13766288; doi:10.1371/journal.pone.0353734)
Supplement: Supplementary file 2 [file pone.0353734.s002.docx]

**S1 Table. In operation and operable nuclear reactors of Japan.** Owner, plant, reactor type, output (MWe), year of commercial operation, age, and current status of the 33 operable units. Updated as of April 2024. Source: World Nuclear Association.

| **Owner** | **Plant Name** | **Reactor Type** | **Output MWe** | **Commercial operation** | **Age** | **Current status** |
| --- | --- | --- | --- | --- | --- | --- |
| JAPC | TOKAI-2 | BWR | 1,110 | 1978 | 45 | Outage (2011.03.11 ~ ) |
|  | TSURUGA-2 | PWR | 1,160 | 1987 | 37 | Outage (2011.05.07 ~ ) |
| Hokkaido EPC | TOMARI-1 | PWR | 579 | 1989 | 34 | Outage (2011.04.22 ~ ) |
|  | TOMARI-2 | PWR | 579 | 1991 | 33 | Outage (2011.08.26 ~ ) |
|  | TOMARI-3 | PWR | 912 | 2009 | 14 | Outage (2011.05.05 ~ ) |
| Tohoku EPC | ONAGAWA-2 | BWR | 825 | 1995 | 28 | Outage (2010.11.06 ~ ) |
|  | ONAGAWA-3 | BWR | 825 | 2002 | 22 | Outage (2011.03.11 ~ ) |
|  | HIGASHIDORI-1 | BWR | 1,100 | 2005 | 18 | Outage (2011.02.06 ~ ) |
| TEPCO | KASHIWAZAKI KARIWA-1 | BWR | 1,100 | 1985 | 38 | Outage (2011.08.06 ~ ) |
|  | KASHIWAZAKI KARIWA-2 | BWR | 1,100 | 1990 | 33 | Outage (2007.07.05 ~ ) |
|  | KASHIWAZAKI KARIWA-3 | BWR | 1,100 | 1993 | 30 | Outage (2007.07.16 ~ ) |
|  | KASHIWAZAKI KARIWA-4 | BWR | 1,100 | 1994 | 29 | Outage (2007.07.16 ~ ) |
|  | KASHIWAZAKI KARIWA-5 | BWR | 1,100 | 1990 | 24 | Outage (2012.01.25 ~ ) |
|  | KASHIWAZAKI KARIWA-6 | ABWR | 1,356 | 1996 | 27 | Outage (2012.03.26 ~ ) |
|  | KASHIWAZAKI KARIWA-7 | ABWR | 1,356 | 1997 | 26 | Outage (2011.08.23 ~ ) |
| Chubu EPC | HAMAOKA-3 | BWR | 1,100 | 1987 | 36 | Outage (2010.11.29 ~ ) |
|  | HAMAOKA-4 | BWR | 1,137 | 1993 | 30 | Outage (2011.05.13 ~ ) |
|  | HAMAOKA-5 | ABWR | 1,380 | 2005 | 19 | Outage (2011.05.14 ~ ) |
| Hokuriku EPC | SHIKA-1 | BWR | 540 | 1993 | 30 | Outage (2011.03.01 ~ ) |
|  | SHIKA-2 | ABWR | 1,358 | 2006 | 18 | Outage (2011.03.11 ~ ) |
| Kansai EPC | MIHAMA-3 | PWR | 826 | 1976 | 47 | Operable |
|  | TAKAHAMA-1 | PWR | 826 | 1974 | 49 | Operable |
|  | TAKAHAMA-2 | PWR | 826 | 1975 | 48 | Operable |
|  | TAKAHAMA-3 | PWR | 870 | 1985 | 39 | Operable |
|  | TAKAHAMA-4 | PWR | 870 | 1985 | 38 | Operable |
|  | OHI-3 | PWR | 1,180 | 1991 | 32 | Operable |
|  | OHI-4 | PWR | 1,180 | 1993 | 31 | Operable |
| Chugoku EPC | SHIMANE-2 | BWR | 820 | 1989 | 35 | Outage (2012.01.27 ~ ) |
| Shikoku EPC | IKATA-3 | PWR | 890 | 1994 | 29 | Operable |
| Kyushu EPC | GENKAI-3 | PWR | 1,180 | 1994 | 30 | Operable |
|  | GENKAI-4 | PWR | 1,180 | 1997 | 26 | Operable |
|  | SENDAI-1 | PWR | 890 | 1984 | 39 | Operable |
|  | SENDAI-2 | PWR | 890 | 1985 | 38 | Operable |
| Total | 33 units |  | 33,083 |  |  |  |
