# Supplementary material for: Transition from fossil fuels to nuclear and renewable energy: Evidence from Japan
Source: PLoS One. 2026 Nov 13;21(11):e0353734. doi: 10.1371/journal.pone.0353734 (PMC13766288; doi:10.1371/journal.pone.0353734)
Supplement: Supplementary file 3 [file pone.0353734.s003.docx]

**S2 Table. Nuclear reactors under construction in Japan.** The three units under construction, with the same fields. Updated as of April 2024. Source: World Nuclear Association.

| **Owner** | **Plant Name** | **Reactor Type** | **Output MWe** | **Commercial operation** | **Age** | **Current status** |
| --- | --- | --- | --- | --- | --- | --- |
| J-power | OHMA | ABWR | 1,383 | TBD | - | Under Construction |
| TEPCO | HIGASHIDORI-1 | ABWR | 1,385 | TBD | - | Under Construction |
| Chugoku EPC | SHIMANE-3 | ABWR | 1,373 | TBD | - | Under Construction |
| Total | 3 units |  | 4,141 |  |  |  |
